# The case for neurons: a no-go theorem for consciousness on a chip

**DOI:** 10.1093/nc/niae037

**Published:** 2024-12-26

**Authors:** Johannes Kleiner, Tim Ludwig

**Affiliations:** Munich Center for Mathematical Philosophy, Ludwig Maximilian University of Munich, Geschwister-Scholl-Platz 1, Munich 80539, Germany; Munich Graduate School of Systemic Neurosciences, Ludwig Maximilian University of Munich, Großhaderner Str. 2, Planegg-Martinsried 82152, Germany; Institute for Psychology, University of Bamberg, Markusplatz 3, Bamberg 96047, Germany; Association for Mathematical Consciousness Science, c/o M3/01.13 Markusplatz 3, Bamberg 96047, Germany; Department of Philosophy, Institute of Technology Futures, Karlsruhe Institute of Technology, Douglasstraße 24, Karlsruhe 76133, Germany; Institute for Theoretical Physics, Utrecht University, Princetonplein 5, Utrecht 3584 CC, The Netherlands

**Keywords:** Artificial Consciousness, Synthetic Phenomenology, Artificial Sentience, Machine Consciousness, No-Go Theorem, Artificial Intelligence, Large Language Model

## Abstract

We apply the methodology of no-go theorems as developed in physics to the question of artificial consciousness. The result is a no-go theorem which shows that under a general assumption, called dynamical relevance, Artificial Intelligence (AI) systems that run on contemporary computer chips cannot be conscious. Consciousness is dynamically relevant, simply put, if, according to a theory of consciousness, it is relevant for the temporal evolution of a system’s states. The no-go theorem rests on facts about semiconductor development: that AI systems run on central processing units, graphics processing units, tensor processing units, or other processors which have been designed and verified to adhere to computational dynamics that systematically preclude or suppress deviations. Whether our result resolves the question of AI consciousness on contemporary processors depends on the truth of the theorem’s main assumption, dynamical relevance, which this paper does not establish.

The question of whether Artificial Intelligence (AI) systems are conscious has emerged as one of critical scientific, philosophical, and societal concern. While empirical support to differentiate theories of consciousness is still nascent and while current measures of consciousness (the simplest example of which is interpretation of verbal reports) cannot justifiably be applied to AI systems, our best hope for reliable answers is to link AI’s potential for consciousness with fundamental properties of conscious experience that have empirical import or philosophical credibility.

Significant progress in this regard has already been achieved at the time of submission of this paper. Chalmers (2023) assesses evidence for or against AI consciousness based on an extensive array of features that a system or organism might possess or lack, such as self-report, conversational ability, general intelligence, embodiment, world or self-models, recurrent processing, or the presence of a global workspace. Wiese (2024) proposes a criterion for distinguishing between conscious and nonconscious AI, anchored in the desiderata of the neuroscientific Free Energy Principle.

In this paper, we propose a result of similar nature, which, however, does not rely on system features and how they relate to consciousness, but on a general property of consciousness: dynamical relevance. Here, ‘dynamical’ refers to the temporal evolution (the dynamics) of a system’s states as described by a theory of consciousness. Consciousness is ‘relevant’ to a system’s time evolution iff the time evolution with consciousness differs from the time evolution without consciousness. Whether consciousness is dynamically relevant depends on the theory of consciousness under consideration and on how far this theory implements consciousness-dependent changes of the dynamical evolution, as compared to a reference theory that addresses the same states.

What sets AI systems apart in the context of consciousness is not the specific computational architecture that is employed; architectures that closely resemble the mammalian brain’s computational structure can arguably also be used, cf. Friston et al. (2022). Instead, the distinctive aspect is the hardware on which an AI architecture operates, namely, Central Processing Units (CPUs), Graphics Processing Units (GPUs), Tensor Processing Units (TPUs), or other processors. This hardware is designed and verified to ensure that the system’s dynamics evolve precisely as described by a computational theory during what is known as functional and postsilicon verification. These verification processes ensure that the design of the chip (the layout of integrated circuits in terms of semiconductors), as well as the actual product (the processing unit after production), yield dynamics exactly as specified by the computational theory. Any dynamical effects that violate the specification of this theory are excluded or dynamically suppressed by error correction.

Our result is an example of a no-go theorem similar to those used in physics. A no-go theorem is a formal theorem that proves a conclusion to hold based on formal assumptions. In our case, these assumptions comprise dynamical relevance of consciousness, as well as formal statements of functional and postsilicon verification.

No-go theorems play an important role in scientific progress in physics. Important examples are Bell’s theorem (Bell, 1964), the Kochen–Specker theorem (Kochen and Specker, 1990), the no-cloning theorem (Wootters and Zurek, 1982; Dieks, 1982), and Earnshaw’s theorem (Earnshaw, 1842), among many others. This role is not necessarily to establish a conclusion beyond doubt, but to direct research and attention to the assumptions that feed into the no-go theorem. Only once such assumptions have been confirmed to hold true, the conclusion of the theorem will be established. (We would like to thank Ryota Kanai for introducing the notion of no-go theorems to consciousness science.)

In this spirit, we too do not contend that our result resolves the issue of AI consciousness. Rather, we take our result to point at the theorem’s assumptions, most notably dynamical relevance, for further research. If dynamical relevance holds true, then our result does have strong implications. If it does not hold true, our result ceases to apply. In explaining our assumption in section ‘Is dynamical relevance plausibly true?’, we do give good reasons for why dynamical relevance may plausibly be true, but our explanations are not intended to establish this beyond reasonable doubt. Rather, they are meant to invite further research to establish clarity with respect to this assumption.

Our theorem is mathematical in nature; it rests on formal quantities and a formal proof. And like formal proofs in other sciences can only be intuitively explained up to a certain point, so can our proof. The following argument is an attempt to explain our proof intuitively, but we would like to stress that this intuition does not capture the result in full. In fact, the objective of formal modelling is to delineate all concepts involved in intuition carefully, so as to present a theorem that underwrites the intuition in both scope and precision.

Verification of processing units ensures that any dynamical effects that change the computational dynamics of a processing unit are precluded or suppressed.
If consciousness is dynamically relevant, and AI systems are conscious, then there are dynamical effects that change the computational dynamics of an AI system.
AI systems run on processing units.
If consciousness is dynamically relevant, AI systems cannot be conscious.

The conclusion (C) follows because qua (A3) and (A1), verification ensures that any dynamical effects that change the computational dynamics of an AI system are precluded or suppressed. (A2) states that if consciousness is dynamically relevant, and AI systems are conscious, then there are dynamical effects that change the computational dynamics of an AI system. Therefore, if consciousness is dynamically relevant, then AI systems cannot be conscious. The crucial work of the formalization we introduce below is to make sure that this reasoning is also sound if consciousness’ dynamical effects apply on a level below the computational level.

In a nutshell, this paper shows that if consciousness makes a difference to how a system evolves in time—as it should if consciousness is to have any evolutionary advantage, e.g.—then any system design which systematically precludes or suppresses diverging dynamical effects systematically precludes or suppresses the system from being conscious.

Before embarking on the formal research that puts the above reasoning on solid ground, we focus on the new concept of dynamical relevance: we explain it in more detail and give reasons for why it may, plausibly, be true.

## What is dynamical relevance?

Dynamical relevance is a formal condition. It is defined in section ‘Dynamical relevance’, once formal preliminaries have been introduced in section ‘Formal preliminaries’. The goal of the present section is to explain and illustrate the concept in non-formal terms, so as to make it accessible to a wide audience.

Dynamical relevance is a relational concept. It describes how something, e.g. a property, relates to the dynamics of a system, as described by a theory. If that ‘something’ is relevant for the dynamics of the system, then we call it ‘dynamically relevant’. In contrast, if that ‘something’ is not relevant for the dynamics of the system, then we call it ‘not dynamically relevant’ or ‘dynamically irrelevant’. Before applying dynamical relevance to consciousness, let us give two examples of how this notion applies to other properties.

### Example 1: A moving car

As an intuitive first example, we consider a hypothetical theory for a moving car. (We thank Wanja Wiese for suggesting this example when discussing our manuscript.) The theory predicts, we presume, how the car behaves as forces are applied to it. In particular, it describes which dynamical trajectory the car takes on a parking lot as forces are applied to its steering wheel and its brake and gas pedals for a given initial position and velocity.

How much load we add to the car is not predicted by the moving-car theory; it requires an extension of this theory that is also capable of dealing with load. If one puts a heavy box into the trunk of the car, the car’s dynamical trajectory will be different from its dynamical trajectory with an empty trunk. This difference might be small and hard to notice or large and easy to notice; for example, in the case of a Moose test, a heavy box in the trunk could make the difference between tipping over and not tipping over. In any case, as the load of the car makes a difference to the dynamics of the car, the moving-car-plus-load theory introduces a new variable that is dynamically relevant with respect to the moving-car theory.

The colour of the car’s seats is also not predicted by the moving-car theory, and if that should be taken into account, an extended model with a new variable that describes said colour is required as well. For example, the seats could be coloured in black, blue, or red. In contrast to the car’s load, however, the moving-car-plus-colour theory will not make changes to the dynamical trajectory of the car; the car’s dynamical trajectory will be the same for all seat colours. Thus, as the seat colour does not make a difference to the dynamics of the car, according to the moving-car-plus-colour theory, the seat colour is dynamically irrelevant with respect to the moving-car theory.

To summarize, for the hypothetical extensions of the moving-car theory outlined earlier, the car’s load is dynamically relevant, whereas the seats’ colour is dynamically irrelevant. We emphasize that the specification of the reference theory is important. With respect to a more elaborate moving-car theory that takes into account the driver and their psychology for the prediction of the car’s dynamical trajectory, the seats’ colour might very well make a difference for the dynamics of the car and, thus, be dynamically relevant.

### Example 2: An electrical circuit

As a more scientific example, we consider an electrical circuit. In an electrical circuit, voltages and charge currents are typically described by electrical circuit theory. For example, Ohm’s law $V = R \cdot I$ relates the voltage drop *V* across an electrical resistor, with resistance *R* to the charge current flow *I* through the resistor. Besides the resistor, the electrical capacitor is another important circuit element. A capacitor stores electrical charge *Q*, when a voltage *V* is applied to it; the capacitor’s capacitance *C* determines the amount of charge that is stored for a given voltage *Q* = *CV*.

Based on the two circuit elements, resistor and capacitor, one can build a simple electrical circuit: a so-called *RC*-circuit, where a capacitor is effectively connected to itself but only via the resistor. When the capacitor is initially charged up to the voltage *V*_0_, it will decay on a timescale $\tau= RC$; explicitly, $V(t) = V_0\, e^{-t/\tau}$. This constitutes a model for the capacitor voltage in an *RC* circuit or, for brevity, RC circuit model.

This model can be extended to take into account further quantities of interest. For example, the resistance of a resistor *R* depends on the temperature *T* of the resistor. Temperature is a concept from thermodynamics but not from circuit theory, so it is not part of the *RC* circuit model as described earlier. But the temperature is relevant for the resistance, and hence it is dynamically relevant for the voltage in an *RC* circuit; it changes how the voltage evolves over time. Thus, a model that extends the *RC* circuit model to take into account temperature posits temperature as dynamically relevant. In contrast, if we extended the *RC* circuit model to take into account the resistor’s colour coating, the new variable would not be dynamically relevant, because the resistor’s colour coating is dynamically irrelevant for the voltage in an *RC* circuit; it makes no difference to how the voltage or other quantities in the original model evolve in time.

### Dynamical relevance of consciousness

Having clarified the concept of dynamical relevance in general contexts, we can now discuss its application in consciousness science. For brevity, we will use the term ‘dynamical relevance’ in what follows to abbreviate the term ‘dynamical relevance of consciousness’.

Dynamical relevance (of consciousness) describes the relation between a theory of consciousness and a reference theory on which the theory of consciousness is built, e.g. a neuroscientific theory that describes those brain functions that operate independently of consciousness. In a nutshell, a theory of consciousness posits consciousness as dynamically relevant, if being conscious makes a difference for the time evolution of a system, as compared to what the reference theory, that does not contain consciousness, would prescribe.

A simple example of a theory of consciousness that posits consciousness to be dynamically relevant is a theory which proposes that consciousness is a specific cognitive function that would be absent if systems did not possess consciousness. Another simple example is a theory of consciousness which posits that consciousness is something nonphysical and endows consciousness with a causal effect on physical states.

### Relation to other properties

Consciousness can be dynamically relevant in both physicalist and nonphysicalist ontologies. That is, it is ontologically neutral. By endorsing dynamical relevance, one is not committed to any specific ontology. As we will now show, dynamical relevance is furthermore implied by other (important) concepts in both physicalist and nonphysicalist contexts. Therefore, dynamical relevance is a weaker assumption than those concepts. It is easier to accept and less demanding than these other concepts.

In physicalist contexts, dynamical relevance is implied by at least three concepts. First, it is implied by strong emergence. That is the case, because the ‘fundamental higher-level causal powers’ (O’Connor, 2021, Section 4), which exist in the case of strong emergence, make a difference to the time evolution of the substrate states.

Second, dynamical relevance can also be implied by some forms of weak emergence. It is arguably implied, e.g., by the information decomposition approach to causal emergence (Mediano et al., 2022). In this approach, even weak emergence induces downward causation. If downward causation implies that there are causal effects of the higher-level property on the lower-level property, then the higher-level property is dynamically relevant to the lower-level property.

Finally, dynamical relevance is also implied by the assumption that consciousness has intrinsic or functional value (Cleeremans and Tallon-Baudry, 2022), which motivates agents and guides their behaviour. That is the case because an agent’s behaviour is part of the agent’s dynamical trajectory. Therefore, if ‘it is only in virtue of the fact that conscious agents ‘experience’ things and ‘care’ about those experiences that they are ‘motivated’ to act in certain ways’ (Cleeremans and Tallon-Baudry, 2022, p. 1), then consciousness is dynamically relevant.

In nonphysicalist contexts, dynamical relevance (of consciousness) is implied by a violation of an ontological assumption known as ‘causal closure of the physical’ or ‘completeness of the physical’ (Robb et al., 2023). This assumption states that for every physical effect, there are sufficient physical causes.

Dynamical relevance is implied by a violation of the causal closure of the physical, because if the physical is not causally closed in virtue of consciousness, there are physical effects at least one of whose jointly sufficient causes is consciousness—usually conceived of as a property or substance separate from the physical properties or substances in this context. But a cause makes a difference to the time evolution of its effect. Hence, it follows that consciousness makes a difference to the time evolution of some physical effects: the time evolution with consciousness differs from what it would have been without consciousness. Thus, if the physical is not causally closed in virtue of consciousness, consciousness is dynamically relevant.

### Is dynamical relevance plausibly true?

Our no-go theorem is predicated on dynamical relevance; it only applies if dynamical relevance holds true, and its conclusions apply to AI systems only in this case.

This paper is not intended to establish dynamical relevance as true. A key function of no-go theorems is to point to the underlying assumptions, and this is exactly what we take the main point of our theorem to be.

What we need to do, however, is to give reasons for why it is plausible to assume dynamical relevance. Some of these reasons have already been given earlier. Because dynamical relevance follows from other assumptions that are taken to be valid—because it is a weaker assumption—it is plausibly true. However, there are also more direct reasons for this, which we review in this section.

Consider, as a simple example, an experiment which relies on a subject’s reports on her conscious experiences. Let us assume that the subject is shown some stimulus followed by a mask and that she has to press a button to indicate whether she has consciously perceived the stimulus, across various trials. Throughout the trials, we might measure her electroencephalogram (EEG) signal, so as to carry out an analysis that distinguishes EEG activity in the case of conscious perception from EEG activity in the case of unconscious perception. This analysis might target a theory of consciousness, so as to confirm or refute whether the difference in EEG signal aligns with the theory’s predictions or retrodictions about this case.

A necessary condition for such an analysis to be possible is that the report—the pressing of a button, in this case—can depend on whether the subject has consciously perceived a stimulus. Put in terms of the theory of consciousness that a study aims at, we may say: a necessary condition, for the aforementioned analysis to be possible, is that the report (or EEG data for that matter) depends on whether the subject is experiencing the stimulus consciously (according to the theory, if it were true). If the time series of reports and EEG data does not depend on consciousness, the experiment cannot have any weight in supporting the theory. In other words, the theory must posit consciousness as relevant to the report or EEG data (or both). And because report and EEG data are part of the dynamics of theories from natural sciences, the theory of consciousness must posit consciousness to be dynamically relevant with respect to these theories. Dynamical relevance is likely a precondition for the experiment and the analysis to work as intended. Further details are needed to cash out this example and to see if it indeed applies. But we take it to show that dynamical relevance is at least plausibly true.

More generally, we may say that any empirical investigation of consciousness relies on measures of consciousness (Irvine, 2013) to infer the state of consciousness of a subject (i.e. some information about the subject’s conscious experience). An experiment may use objective measures of consciousness that rely on behavioural or neural markers, or subjective measures of consciousness that rely on a subject’s reports about their conscious experience. Both types of measures rely on data that are part of the dynamics of the physical. For a measure of consciousness to work as expected—to allow us to infer something about the state of consciousness of a subject—consciousness must make a difference to the data that feed into the measure. It must make a difference to the dynamics that explain the data, and hence be dynamically relevant, with respect to a theory that contains such an explanation.

The same argument can be made not only for scientific investigations but also for any kind of intersubjective exploration of conscious experiences. Debating consciousness relies on certain dynamics of the vocal cord (among many other things), making art about consciousness makes use of behaviour. All these cases are part of the dynamics of an organism, and if the dynamics are to depend on consciousness, consciousness needs to be dynamically relevant. (This argument can be strengthened by considering what is required to distinguish two or more theories of consciousness empirically, cf. Kleiner and Hartmann (2023), where, however, dynamical relevance is referred to as ‘empirical version of the closure of the physical’ and formulated in more generality than we do here.)

The upshot of these arguments is that dynamical relevance could well be a necessary condition for the type of activities we carry out when engaging in empirical scientific studies of consciousness. These arguments do not show that dynamical relevance is true. For all we know, there is the possibility that it is not. But if it is not, the empirical investigation of consciousness—and with it the science of consciousness—might not make sense; a necessary condition for its possibility would likely be violated.

### Current theories

The aforementioned arguments do not depend on any specific theory of consciousness. But it is interesting to ask what current theories of consciousness say about dynamical relevance.

First, it is important to note again that empirical tests of theories of consciousness presume that consciousness is dynamically relevant according to these theories. That is the case, because they assume that whatever is measured can corroborate or falsify a theory, or speak in favour of one theory rather than another. For this to be possible, consciousness must make a difference to the data. Because the data are drawn from the physical dynamics of a system, consciousness must be dynamically relevant.

Second, we can consider the metaphysics of theories of consciousness. In cases where these are clear, they do, in our eyes, imply dynamical relevance. Consider, as an example, Integrated Information Theory (IIT) (Oizumi et al., 2014). IIT assumes that experience is primary and physics—or better, physical descriptions—are secondary. In a sense, only experience exists in the form of cause-effect structures. Hence, it should be the case that experience makes a difference to the physical dynamics, so that conscious experience is dynamically relevant.

Another example is Global Neuronal Workspace Theory (GNW) (Dehaene et al., 2011). Here, too, we think, the metaphysical interpretation implies dynamical relevance. GNW assumes that conscious experiences are tied to a global neuronal workspace, ‘consisting of a distributed set of (...) neurons characterised by their ability to receive from and send back to homologous neurons in other (...) areas horizontal projections through long-range excitatory axons’ (Dehaene et al. (2011), p. 56). Organisms that possess a workspace are conscious, while organisms that do not possess a workspace are not conscious, according to the theory. Hence, whether a system is conscious makes a difference to a system’s information processing architecture and, *a fortiori*, to the system’s dynamics.

The only thing which speaks against dynamical relevance among current theories of consciousness, in our eyes, is their mathematical formulation (in those very limited cases where a mathematical formulation exists).

Consider, e.g., IIT. The mathematics of IIT is given in terms of an unwieldy algorithm that takes as an input a physical description of a system, as given by some reference theory, and provides as output a mathematical description of the conscious experience of that system. An analysis of the mathematics that underlie this algorithm shows that the algorithm defines a map which goes from the physical description of a system to the descriptions of conscious experience (Kleiner and Tull, 2021; Tull and Kleiner, 2021).

Therefore, according to IIT’s mathematics, consciousness is not dynamically relevant. The physical evolution of the systems is exactly as they are in the reference theory that provides the input to IIT. No change whatsoever is introduced to these dynamics by the theory. Hence, the mathematics of IIT do not instantiate dynamical relevance.

In our view, this is an issue of the mathematical formulation that IIT applies. The mathematics do not naturally align with the metaphysical foundation of the theory, and the exact same formal properties which speak against dynamical relevance are the source of other issues, most notably issues with falsifying the theory, cf. Kleiner and Hoel (2021), and issues related to the unfolding argument, more generally (Doerig et al. (2019)). The mathematics of IIT may need to be revised, at the very least to instantiate dynamical relevance, so as to resolve these problems with falsification.

### Definitions

We conclude this section with a pointer to the places in the manuscript where the precise definition of dynamical relevance is given: in the ‘Dynamical relevance’ section, Definitions 1 and 2. Definition 1 is epistemic. It defines the concept of dynamical relevance with respect to a theory of consciousness, relative to some underlying neuroscientific theory, independent of whether either of the theories is true. Definition 2 then builds on this epistemic definition to provide an ontic definition. This definition is about whether consciousness is actually dynamically relevant. What is crucial in Definition 2 is that it suffices that there is ‘some’ reference theory with respect to which the true theory of consciousness satisfies Definition 1. This is sufficient to prove our result, Theorem 4. Referencing the actual world is important in the context of this result because postsilicon verification is about what actually happens, once a processing unit has been manufactured.

## No-go theorem

### Formal preliminaries

The central notion which underlies our result is that of the time evolution of a system’s states. Given a scientific theory *T* and a system *S* within the scope of the theory, we denote by $k_T(S,s)$ the dynamical evolution (also called ‘trajectory’) of *S* with initial state *s*. This dynamical evolution describes how the state *s* evolves in time according to *T*. An example is the evolution of a brain state according to a neuroscientific theory. We will abbreviate $k_T(S,s)$ by *k_T_* if it is clear from context that we are talking about one system and one initial state.

The class of scientific theories which is relevant in the present context are theories of consciousness, on the one hand, and scientific theories on which theories of consciousness are built, on the other hand. These scientific theories are theories that a theory of consciousness makes use of to explain how consciousness relates to the brain, and to which it refers for all explanations that do not involve consciousness: the theories that have been developed in neuroscience or other natural sciences. We use the symbol ϒ to denote all such theories that are relevant for AI or consciousness and refer to the theories in this class as ‘reference theories’, because they are the theories that a theory of consciousness can refer to. Examples are theories of neuroscience, biology, chemistry, computer science, and physics.

Different theories describe systems at different levels (List, 2019), and in some cases, the states of a system posited by one theory *T* (the ‘lower’ level) can (in principle) be mapped to states of another theory *T*ʹ (the ‘higher’ level). If this is the case, we write $T < T^{\prime}$. Because dynamical evolutions are sequences of states, if $T < T^{\prime}$, we can map any dynamical evolution $k_T(S,s)$ of *T* to a (not necessarily dynamical) evolution of *T*ʹ, which we denote as $k_T (S,s)|_{T^{\prime}}$. In cases where $T$ and $T^{\prime}$ are reference theories, we assume that any dynamical evolution is mapped to a dynamical evolution (with the corresponding initial state: $k_T(S,s)|_{T^{\prime}} = k_{T^{\prime}} (S,s|_{T^{\prime}})$).

We assume that there is a reference theory $T_\text{F} \in \Upsilon$ that can be mapped to states of any other reference theory in ϒ, which means that $T_\text{F} < T $ for all $T \in \Upsilon$. For lack of a better term, we will refer to this theory as a ‘fundamental reference theory’, but emphasize that it does not have to be ‘the true’ fundamental theory. The requirement that $T_\text{F} < T $ for all $T \in \Upsilon$ is only an epistemic requirement that expresses relationships between theories in ϒ, and leaves open whether *T*_F_, or any other theory in ϒ for that matter, is the true theory which correctly describes the actual dynamics. Whether this can be the case depends precisely on the question of whether consciousness is dynamically relevant. What justifies the assumption that there is a theory whose states can be mapped to states of the other theories (whose states ‘ground’ the states of all other theories, one might say) is that the states of quantum theory can, in principle, be mapped to states of all physical theories in ϒ. That is because quantum theory is what underlies condensed-matter theories as far as they are relevant for semiconductors and integrated-circuit design of processors. So, for all practical purposes, we can think of *T*_F_ as quantum theory.

Finally, we assume that there is a fact to the matter of what the real (that is: actual) dynamics of any system are, even if that fact may not be knowable. We denote the description of the real dynamics in terms of the states of any reference theory $T \in \Upsilon$ (any ‘level’ of description, so to speak) by $k^\ast|_T$. If $T\ltT^{\prime}$, the description of the real dynamics in terms of the states of both theories are compatible, i.e. $k^\ast|_T|_{T^{\prime}} = k^\ast|_{T^{\prime}}$.

### Dynamical relevance

Theories of consciousness (ToCs), sometimes also called models of consciousness, express a relation between a description of a system, on the one hand, and a description of its conscious experiences, on the other hand. The latter could be a description of its phenomenal character (cf., e.g. Lee (2021), Kleiner and Ludwig (2024)) or simply an expression of whether a system *S* has conscious experiences at all. We now expand the formalism introduced in the last section to take this into account.

Together, the description of a system and the description of its conscious experience constitute a state *s* of the ToC. Because a ToC expresses a relation between a description of a system and a description of its conscious experiences, the state *s* contains both a nonexperiential and an experiential part, which we refer to as the ‘reference state’ and ‘state of consciousness’, to have a simple terminology that is free of metaphysical burden. The dynamical evolution $k_M(S,s)$ of a system *S* in a state *s* of the theory/model of consciousness *M* expresses how the reference state and the state of consciousness relate according to the theory.

Because ToCs contain a reference description of a system at some level, for every toc *M*, there is at least one reference theory $T_\text{R} \in \Upsilon$ such that the physical part of any state *s* of *M*, and therefore also any dynamical evolution *k_M_*, can be expressed in *T_R_*. We denote this state by $s|_{T_\text{R}}$ and the expression of the reference part of the trajectory *k_M_* in terms of *T*_R_ by $k_M|_{T_\text{R}}$. So, $k_M|_{T_\text{R}}$ is what *M* says about the evolution of reference states on *T*_R_’s level of description. We call any such *T*_R_ an ‘underlying’ reference theory of *M*.

To offer an alternative perspective that might be helpful to illustrate this notation, consider again that any theory of consciousness *M* expresses a relation between a description of a system and a description of its conscious experiences, or if framed in the terminology we have just introduced: a relation between a reference state and a state of consciousness. Let us suppose that the former constitute a set $\tilde P$ and that the latter constitute a set *E*. Here, we are adding a ‘~’ on top of *P* because the states which the theory of consciousness uses might not be identical to the states that any reference theory uses; there could be simplifications, for example. What needs to be the case, however, is that these states can be mapped to the states of some reference theory *T*_R_. The states of the reference theory are what the theory of consciousness ‘means’ when addressing reference states, so to speak. Let us assume that the reference states of *T*_R_ form a set. A trajectory *k_M_* of *M* is a trajectory over $\tilde P \times E$. By restricting to $\tilde P$ and then mapping to *P*, we obtain a trajectory over *P*. This is what the symbol $k_M|_{T_\text{R}}$ denotes: it is what the trajectory of *M* implies for the time evolution as expressed in terms of the states of the reference theory *T*_R_.

Independently of the description that a ToC applies on the side of consciousness, there is a fact to the matter of whether a system is conscious when in a trajectory $k_M(S,s)$. This means: whether the system *S* has conscious experiences at least at one point of time in the dynamical evolution $k_M(S,s)$. Making use of the important link between ToCs and reference descriptions, we can say that a system *S* is conscious in a dynamical trajectory $k_{T_R}$ of the reference theory iff there is a dynamical evolution *k_M_* of *M* such that (i) we have $k_M|_{T_\text{R}} = k_{T_\text{R}}$ and (ii) the system is conscious in *k_M_*.

Whether a ToC has anything original to say about the dynamical evolution of its reference states, or simply presumes the dynamical evolution of a reference theory—of an underlying neuroscientific theory, that is, in most cases—is precisely the question of dynamical relevance, defined as follows. Let *M* denote a ToC and $T_\text{R} \in \Upsilon$ a reference theory thereof.

Definition 1. Consciousness is dynamically relevant according to *M* with respect to *T_R_* iff

$$ S\ \textrm{ is conscious in } k_M \ \Rightarrow \ k_M|_{T_\text{R}} \neq k_{T_\text{R}} \:. $$

Here, the right-hand side is short-hand for $k_M(S,s)|_{T_\text{R}} \neq$  $k_{T_\text{R}}(S,s|_{T_\text{R}})$, where $s|_{T_\text{R}}$ denotes the restriction of the state *s* of *M* to *T*_R_. The left-hand side is a shorthand for ‘*S* is conscious in $k_M(S,s)$’, meaning that there is at least one point of time in $k_M(S,s)$ so that *S* has a conscious experience at that time according to *M*. The condition has to hold for all dynamical trajectories *k_M_* of *M*, meaning for the dynamical trajectories of all systems *S* in the scope of *M* and all states *s* of these systems.

This definition expresses the intuition that if *S* is conscious according to a ToC *M*, then the dynamical evolution as specified by *M* differs from the dynamical evolution as specified by the underlying neuroscientific theory alone.

We have already referenced the ‘real’ dynamics of a system and introduced the symbol $k^\ast|_{T_\text{R}}$ to denote what the real dynamics of a system would look like in terms of the states of *T*_R_. There is also a fact to the matter of whether a system in a trajectory $k^\ast$ is conscious and how conscious experiences relate to the physical. That is, there is a ‘true’ or ‘real’ theory of consciousness, which we denote by $M^\ast$. As in the physical case, $M^\ast$ may be unknown or unknowable. We will denote its dynamical evolutions by $k_{M^\ast}$. Because these describe what really happens, we have $k_{M^\ast}|_{T_\text{R}} = k^\ast|_{T_R}$ for all *T*_R_. Using $M^\ast$, we can define dynamical relevance simpliciter:

Definition 2. Consciousness is dynamically relevant (CDR) only if it is dynamically relevant according to the ‘true’ ToC $M^\ast$ with respect to some reference theory $T_\text{R} \in \Upsilon$.

### Functional and postsilicon verification

What is unique about AI systems in the present context is not the particular architecture that is employed; AI can also be built on architecture derived from the brain; cf., e.g.  Friston et al. (2022). What is unique is rather that the architecture runs on CPUs, GPUs, TPUs, or other processors that have been designed and verified in the lab.

There are two major verification steps in processor development, called functional and postsilicon verification. Functional verification (Mishra and Dutt, 2005; Wile et al., 2005) is applied once the design of a processor in terms of integrated circuits has been laid out, but before the manufacturing phase begins. It applies simulation tools, formal verification tools, and hardware emulation tools to ensure that the design of the chip meets the intended specifications as described by a computational theory ${T_{\textrm{comp}}}$. In almost all cases, the computational theory is Gödel–Church–Turing computation, with the particular functions that are computed specified by a system’s Instruction Set Architecture.

Postsilicon verification (Mishra et al., 2017; Mitra et al., 2010) is applied after the silicon waver has been fabricated. It applies in-circuit testing, functional testers, failure analysis tools, and reliability testing, among other things, to ensure that the physical product works as ${T_{\textrm{comp}}}$ would have it. Present-day examples of the theory ${T_{\textrm{comp}}}$ are the ARM Instruction Set Architectures on which most data centre servers run or the X-86 Instruction Set Architecture on which most desktop devices run.

Functional verification is a theoretical endeavour: it applies simulation and emulation tools based on a theoretical account on how the substrate, on which a processor is to be built, behaves. Because this substrate is a semiconductor, this theoretical account is based on quantum theory. Put in terms of dynamics, functional verification aims to ensure that whatever happens in the quantum realm, or below, implements or is compatible with the dynamics as described by ${T_{\textrm{comp}}}$, formally:


(1) $$ k_{T_\text{F}}|_{{T_{\textrm{comp}}}} = k_{T_{\textrm{comp}}} $$

for all dynamical evolutions of a processor *S*. This condition could fail, e.g. because of leakage currents, most notably those created by tunnelling of electrons through a transistor’s gate oxide layer. Tunnelling is an effect described by quantum theory and needs to be controlled for in order to ensure transistors that implement a chosen ${T_{\textrm{comp}}}$.

Postsilicon verification, on the other hand, is applied to a chip once it has been built. It ensures that the dynamics of the actual physical product comply with ${T_{\textrm{comp}}}$. Making use of the $k^\ast$ notation to denote the actual dynamical evolution of a system, postsilicon verification enforces that


(2) $$ k^\ast|_{{T_{\textrm{comp}}}} = k_{T_{\textrm{comp}}} $$

for all dynamical evolutions of a processor *S*.

Being an AI system means running on CPUs, GPUs, TPUs, or other processors that have been designed and verified. That is what makes the system ‘artificial’. And because processor dynamics compose (the output of one is the input of the next), verification holds for AI systems as well: there is an underlying computational theory ${T_{\textrm{comp}}}$ that accounts for what ‘happens’ on the processors, while the system is running, and the computational dynamics satisfy (1) and (2).

### AI consciousness

With all this in place, we can formulate the question that is being asked precisely. The term ‘Artificial Intelligence’ is used very broadly, comprising many different computational architectures and applications. What one means when one asks whether an AI system is conscious is whether the computational architecture that is applied by this system, with the specific quirks of its implementation and training, potentially in a specific task, has conscious experiences. The architecture and these specifics determine the computational dynamics the system is capable of. Thus, the question is whether the system has a computational evolution $k_{T_{\textrm{comp}}}$ such that it is conscious in this computational evolution according to a theory of consciousness *M*; cf. section ‘Dynamical relevance’ for a definition of what this means in terms of dynamics *k_M_* of *M*. In summary:

Definition 3. An AI system S is conscious according to a theory of consciousness *M* only if there is at least one dynamical evolution $k_{T_{\textrm{comp}}}$ in which the system is conscious according to *M*.

This is a very weak condition, which, however, has one important consequence: that the question of AI consciousness is determined by facts on the computational level and above; it is independent of what happens on a subcomputational level. That is, if we have a trajectory $k_{T_\text{R}}$ on a subcomputational level ($T_\text{R} < {T_{\textrm{comp}}}$) with $k_{T_\text{R}}|_{{T_{\textrm{comp}}}} = k_{{T_{\textrm{comp}}}}$, then *S* is conscious in $k_{T_{\textrm{comp}}}$ only if it is conscious in $k_{T_\text{R}}$.

### No-go theorem

Our main result is the following theorem.

Theorem 4. If consciousness is dynamically relevant, then AI systems are not conscious.

Before giving the proof, we first illustrate the result for the simpler case where consciousness is dynamically relevant with respect to the computational level ${T_{\textrm{comp}}}$ itself. The power of the theorem is to extend this result to all other cases. Subsequent to this illustration, we prove a lemma needed for the main theorem and then proceed to prove the theorem itself.

So let us consider the case where *T*_R_ in Definition 2 is ${T_{\textrm{comp}}}$. That is, the following chain of reasoning assumes that consciousness is dynamically relevant (Definition 2) with respect to ${T_{\textrm{comp}}}$.

Let *S* be an AI system. Because of postsilicon verification (2), all of the dynamical evolutions of *S* satisfy


(3) $$ k^\ast|_{{T_{\textrm{comp}}}} = k_{T_{\textrm{comp}}} \:. $$

Application of Definition 2 for the case $T_\text{R} = {T_{\textrm{comp}}}$ implies, via Definition 1, that if *S* is conscious in a $k_{M^\ast}$, then $k_{M^\ast}|_{{T_{\textrm{comp}}}} \neq k_{{T_{\textrm{comp}}}}$. The converse of this statement is that if $k_{M^\ast}|_{{T_{\textrm{comp}}}} = k_{{T_{\textrm{comp}}}}$, then *S* is not conscious in $k_{M^\ast}$. From the paragraph before Definition 2, we have $k_{M^\ast}|_{T_\text{R}} = k^\ast|_{T_\text{R}}$ for all *T*_R_. Setting $T_\text{R} = {T_{\textrm{comp}}}$, this gives $k_{M^\ast}|_{{T_{\textrm{comp}}}} = k^\ast|_{{T_{\textrm{comp}}}}$, which is why the identity (3) establishes the prerequisite of the above condition for all dynamical evolutions of *S*. Therefore, it follows that *S* is not conscious in any $k_{M^\ast}$. Thus, Definition 3 implies that *S* is not conscious, as claimed.

The remainder of this section is devoted to the proof of the theorem in the general case. To this end, we first state and prove the following lemma.

Lemma 5. Dynamical relevance passes downward, in the sense that if $T_\text{R} < T_\text{R}^{\prime}$ and consciousness is dynamically relevant according to *$M^\ast$* with respect to $T_\text{R}^{\prime}$, then it is also dynamically relevant according to *$M^\ast$* with respect to *T*_R_.

Proof of the Lemma: Consciousness is dynamically relevant according to *$M^\ast$* with respect to $T_\text{R}^{\prime}$, iff

$$S\;\text{is conscious in}k_{M^\ast}\;\Rightarrow\;k_{M^\ast}\vert_{T_\text{R}^{\prime}}\neq k_{T_\text{R}^{\prime}}\:.$$

Because $T_\text{R} < T_\text{R}^{\prime}$, there is a function which maps states and dynamical evolutions from *T*_R_ onto $T_\text{R}^{\prime}$. Furthermore, we have $\textstyle k_{M^\ast}\vert_{T_R}\vert_{T_R^{\prime}}=k^\ast\vert_{T_R}\vert_{T_R^{\prime}}=k^\ast\vert_{T_R^{\prime}}=k_{M^\ast}\vert_{T_R^{\prime}}$. Therefore, it follows that


$$k_{M^\ast}\vert_{T_\text{R}^{\prime}}\neq k_{T_\text{R}^{\prime}}\;\Rightarrow\;k_{M^\ast}\vert_{T_\text{R}}\neq k_{T_\text{R}}\:.$$

Together with the above, this gives


$$S\;\text{is conscious in}k_{M^\ast}\;\Rightarrow\;k_{M^\ast}\vert_{T_\text{R}}\neq k_{T_\text{R}}\:,$$

which is the case iff consciousness is dynamically relevant according to $M^\ast$ with respect to *T*_R_.

We now proceed to the proof of the theorem.

Proof of the Theorem: We first consider the case where *T*_R_ in Definition 2 is *T*_F_.

Let *S* be an AI system. Because of functional and postsilicon verification, we have

(4) $$k_{T_\text{F}}\vert_{T_\text{comp}}=k_{T_\text{comp}}=k^\ast\vert_{T_\text{comp}}$$

for all dynamical evolutions of *S*. Because consciousness is (by assumption) dynamically relevant and we have assumed $T_\text{R} = T_\text{F}$, Definition 1 applies to give

(5) $$ S\ \textrm{ is conscious in } k_{M^\ast} \ \Rightarrow \ k_{M^\ast}|_{T_\text{F}} \neq k_{T_\text{F}} $$

for all dynamical trajectories $k_{M^\ast}$ of $M^\ast$.

Let us now assume that *S* is conscious in some trajectory $k_{M^\ast}$ of $M^\ast$. According to the last implication, we thus have

$$ k_{M^\ast}|_{T_\text{F}} \neq k_{T_\text{F}} \:. $$

Because $T_\text{F} < {T_{\textrm{comp}}}$, we can map both these trajectories to ${T_{\textrm{comp}}}$. For $k_{M^\ast}|_{T_\text{F}}$, this gives

$$\begin{aligned} k_{M^\ast}|_{T_\text{F}}|_{{T_{\textrm{comp}}}} &= k^\ast |_{T_\text{F}}|_{{T_{\textrm{comp}}}} \\ &= k^\ast|_{{T_{\textrm{comp}}}} = k_{M^\ast} |_{{T_{\textrm{comp}}}} \:, \end{aligned}$$

where we have made use of identities established in the sections ‘Formal preliminaries’ and ‘Dynamical relevance’. Equation (4) furthermore establishes that

$$ k_{M^\ast} |_{{T_{\textrm{comp}}}} = k^\ast|_{{T_{\textrm{comp}}}} = k_{T_{\textrm{comp}}} \:. $$

The two facts that (i) $k_{M^\ast}|_{{T_{\textrm{comp}}}} = k_{T_{\textrm{comp}}}$ and (ii) that *S* is conscious in $k_{M^\ast}$ establish that *S* is conscious in $k_{T_{\textrm{comp}}}$.

Equation (4) also establishes that

$$ k_{T_\text{F}}|_{{T_{\textrm{comp}}}} = k_{T_{\textrm{comp}}} \:. $$

Because of this equation and $T_\text{F} < {T_{\textrm{comp}}}$, the implication of Definition 3 explained in the last paragraph of the section ‘AI consciousness’ applies and establishes that *S* is conscious in $k_{T_\text{F}}$.

Unwrapping what ‘*S* is conscious in $k_{T_\text{F}}$’ means by definition, we find that there must be a dynamical evolution $\tilde k_{M^\ast}$ of $M^\ast$ such that

$$\begin{aligned} &\textrm{(i) } \tilde k_{M^\ast}|_{T_\text{F}} = k_{T_\text{F}} \quad \textrm{and}\\ &\textrm{(ii) } S\ \textrm{ is conscious in } \tilde k_{M^\ast} \:. \end{aligned}$$

Together, these two conditions violate (5). Thus, we have arrived at a contradiction.

The assumptions that went into the derivation of this contradiction were that consciousness is dynamically relevant with respect to the *T*_F_ level, that *S* is an AI system, and that *S* is conscious in a trajectory $k_{M^\ast}$ of *M*. The first assumption is stated as a condition in the theorem. Thus, it follows that the latter two cannot be both the case.

Because $k_{M^\ast}$ was arbitrary, it follows that an AI system *S* cannot be conscious in any trajectory $k_{M^\ast}$ of $M^\ast$. Consequently, applying Definition 3, it cannot be conscious at all. This establishes the claim that if consciousness is dynamically relevant with respect to *T*_F_, then AI systems are not conscious.

It remains to consider all other cases of *T_R_* in Definition 2. Therefore, let us assume that consciousness is dynamically relevant with respect to some $T_\text{R} \neq T_\text{F}$. Because $T_\text{F} < T_\text{R}$ for all $T_\text{R} \in \Upsilon$ and because dynamical relevance passes downward (Lemma 5), it follows that consciousness is also dynamically relevant with respect to *T*_F_. Hence, the previous case applies and the result follows in full generality.

## Objections

In this section, we discuss a few immediate responses to our result.

### Verification is imperfect

Verification is an industrial process that may not be perfect: despite functional and postsilicon verification, the actual dynamics of a processor may not adhere to the computational theory targeted by verification in all cases. Verification may leave a bit of wiggle room for the dynamics to diverge from the computational theory. Could this wiggle room suffice for consciousness to unfold its dynamical effects?

Any answer to this question depends on how exactly consciousness is dynamically relevant and which imperfections arise in day-to-day verification. It is natural to expect that consciousness’ dynamical relevance is systematic in nature: dynamical effects should systematically occur if a system is conscious and make a systematic difference to how the system evolves in time. The imperfections in day-to-day verification, on the other hand, are likely to be mostly random in nature, meaning that the deviations in dynamical evolution they fail to suppress are random too, both in time (when a deviation can occur) and in the extent to which they can make a difference. If this is true, it is unlikely that the wiggle room left open due to imperfections suffices for consciousness to unfold its dynamical effects.

### Determinism

One objection to our result takes our result to show or imply that a deterministic system cannot be conscious and argues that this is very unlikely to be true. Hence, the result must be wrong or rest on very weak assumptions, so the objection goes.

This objection fails because our result does not show or imply that deterministic systems cannot be conscious. What prevents a system from being conscious, according to our result, is that its design forces it to comply to a formal system that is independent of consciousness. The system is ‘locked into’ a formal system, so to speak. It cannot deviate from it. Reality is forced to adhere to a theoretical construct, by design.

Our result is fully compatible with deterministic systems and also with a deterministic relevance of consciousness to a system’s dynamics.

### Probabilistic processing

Verification as applied in industry targets deterministic computational theories. Would our result also hold in the case of verified probabilistic processing?

The mathematical framework we apply is compatible with probabilistic processing: we do not make an assumption as to whether the notions of state and dynamical evolution are deterministic; a state may well be a probability distribution, and its dynamical evolution a stochastic process. Verification, in this case, implies that a system conforms to the stochastic process as described by a stochastic computational theory. This leaves room for consciousness to have a dynamical effect, but only if this effect conforms to the probability distributions as described by the stochastic computational theory. That is, consciousness may determine how the probability distributions of the stochastic computational theory are sampled, but it cannot change them. As in the case of imperfect verification, we remain sceptical as to whether this limited freedom is compatible with the systematic nature of consciousness’ dynamical effects that are to be expected.

### Quantum computing

Does our result also hold true in the case of quantum computing? Quantum computing is a young industry, and it is not yet clear which type of verification, if any, will need to be deployed. It is likely, however, that any type of verification will need to presuppose a notion of ‘measurement’, which is an inherently vague concept in quantum theory (Bell, 1990) that is partially external to the account of quantum dynamics by the Schrödinger equation. If consciousness were related to measurement (e.g. via consciousness-induced dynamical collapse as proposed in Chalmers and McQueen (2024), then verification might leave enough room for consciousness to have a systematic and meaningful effect. If, on the other hand, consciousness is not related to measurement in quantum theory, it is likely that verification of quantum computers to adhere to quantum dynamics will preclude any potential dynamical effects of consciousness, just as in the classical case.

### Consciousness entailed by particular physical states

A final objection to our result concerns views according to which consciousness is entailed by particular physical states, e.g. substrate-dependent views of consciousness, such as biological naturalism (Seth, 2024). The worry is that such views postulate consciousness as dynamically relevant (in some sense). So should not our result also apply to those views and imply, counter to fact, that the particular physical states these views pick out cannot be conscious? (We would like to thank an anonymous reviewer for raising this point.)

This objection is centred around the following two questions, which we now answer in turn:

- Which implications do our results have for such views?
- How does the concept of dynamical relevance relate to these views?

For most, if not all, substrate-dependent views, and in particular biological naturalism, the answer to question (i) is none. This is the case because our result presumes verification: it only applies to systems that run on a substrate that is verified (cf. section ‘Functional and postsilicon verification’). But most, if not all, substrate-dependent views of consciousness target biological substrates, where no process akin to verification exists. Hence, our result does not apply to these systems. Theorem 4 only establishes the conclusion that AI systems aren’t conscious, where AI systems are systems that run on CPUs, GPUs, TPUs, or other processors that ‘have been designed and verified in the lab’ (p. 10). The theorem does not apply to systems with other substrates.

This limitation in the scope of our result may be obscured by the fact that both artificial and biological systems may be viewed as carrying out computations; but there is a substantial difference in how they carry out computations. Biological systems compute, but they realize a different type of computation than contemporary AI systems. There are reasons to think that this type of computation is mortal computation, as proposed in Hinton (2022), cf. Kleiner (2024) and Seth (2024).

This answer to question (i) is independent of whether substrate-dependent views, or other views according to which consciousness is entailed by particular physical states, postulate consciousness as dynamically relevant (question (ii)). But thinking about the latter question helps to clarify the concept of dynamical relevance, which is why we would like to answer question (ii) as well.

One way to provide an intuition about the technical definition of dynamical relevance that we apply in this paper is to use the concept of ‘making a difference’: roughly speaking, one could say that consciousness is dynamically relevant iff it makes a difference. Because substrate-dependent views of consciousness may claim that a substrate configuration cannot arise without consciousness, so that consciousness is in fact some form of enabling condition that makes a difference, this raises the question of whether these views imply that consciousness is dynamically relevant? This is what motivates question (ii).

We would like to provide two responses to this question. First, we would like to point out that the notion of making a difference, when used in this generality, does not provide a good intuition for what dynamical relevance is about. Dynamical relevance, as used here, is about whether consciousness ‘makes a difference to how a system evolves in time’ (p. 3), ‘a difference for the time evolution of a system’ (p. 5). Cases in which a property is an enabling condition, or in which there is a metaphysical grounding relation, are not included in the definition of dynamical relevance as proposed here (Definition 1). Hence, substrate-dependent views do not imply dynamical relevance.

Rather, and this is the second response we would like to provide, substrate-dependent views can have both dynamical relevant and dynamical irrelevant flavours. We think that this is an important point that sheds light on why dynamical relevance is a decisive assumption, which is why we would like to explain this point in more detail.

Much of the contemporary discussion around consciousness is centred around the relation that holds between consciousness, on the one hand, and the subject matter of the natural sciences, on the other hand. Views where ‘consciousness is entailed by particular physical states’, e.g., postulate a relation between states of consciousness, on the one hand, and substrate states, on the other hand. It is helpful to express this formally. Denoting states of consciousness by $\mathcal C$ and substrate states by $\mathcal P$, the view expresses constraints about a relation


$$ R \subset \mathcal P \times \mathcal C \:. $$

(The exact constraint depends on what one takes the term ‘entail’ to mean. The constraint could, e.g., be that the relation *R* is a function from $\mathcal P$ to $\mathcal C$.)

Dynamical relevance, in contrast, does not restrict the relation that states of consciousness and substrate states have, but rather how these states co-evolve in time. It says something about whether the time evolution of the $\mathcal C$-states is relevant for (‘makes a difference to’) the time evolution of the $\mathcal P$-states.

That is, the notion of dynamical relevance is, to some degree, orthogonal to many of the questions that are being targeted in contemporary discussions: whether consciousness is dynamically relevant is not fixed by one’s assumptions about the relation *R*. Rather, whether dynamical relevance holds is dependent on assumptions that have to be made in addition to those that concern *R*. One can be a biological naturalist and hold that ‘consciousness is a property of only, but not all, living systems’ (Seth, 2024) with or without assuming dynamical relevance in addition. (That is not to say that dynamical relevance is orthogonal to contemporary problems. It is intimately related to both metaphysical assumptions like causal closure and contemporary theories of consciousness, cf. sections ‘Relation to other properties’ and ‘Current theories’.)

## Conclusion

This paper addresses the question of whether AI systems are conscious. Its objective is to introduce a new formal tool, in the form of a no-go theorem, that may provide an answer to this question which is independent of the specific computational architecture that an AI system utilizes and which does not rely on any specific cognitive feature that an AI system might possess or lack.

The no-go theorem is based on what we take to be the only property that distinguishes AI systems from other cognitive systems, a property that might well embody the actual meaning of the word ‘artificial’ in Artificial Intelligence: that the system runs on a substrate that has been designed and verified, rather than naturally evolved.

Ultimately, we believe that any scientific statement about whether a system is conscious needs to be based on a theory of consciousness that is supported by theoretical, philosophical, and, most importantly, empirical evidence. Consciousness science searches for such theories. The crucial premise in our result—dynamical relevance—is a property which theories ascribe to consciousness, so that our theorem can be regarded as establishing a fact about AI’s capability for consciousness for a whole class of theories of consciousness: all those that posit consciousness to be dynamically relevant. Results of this form are important as long as evidence in favour of any single theory of consciousness, as well as evidence to distinguish among them, is still in its early stages, and while the space of possible theories remains only partially explored.

Our result has a few interesting, slightly funny, and potentially relevant implications for AI engineering and AI interpretability. The most notable of these is that our result shows that if an AI system states that it is conscious, then this cannot be because it is conscious. That is to say, even if an AI system were conscious, the cause of any such statement cannot be that the AI system is conscious. This follows because if such a cause existed, consciousness would have to be dynamically relevant, in which case our theorem implies that the system is not conscious. Another implication is that if consciousness has functions that could improve a system’s information processing, then, to make use of those functions, theories of consciousness should be taken into account when designing the substrate on which an AI system will run.

The question of whether AI systems are conscious is of major societal concern (Association for Mathematical Consciousness Science, 2023). It has important ethical (Bostrom and Yudkowsky, 2018; Metzinger, 2021), legal (Benzmüller and Lomfeld, 2020; Susskind, 2019), and technological consequences and will likely play a major role in shaping governance of AI and how individuals interact with this technology. Our result aims to deliver a rigorous and justified answer to this question that does not rely on particular assumptions, such as the truth of a particular theory of consciousness or the validity of a particular test of consciousness when applied to AI systems. The result relies on the truth of its main assumption, dynamical relevance, further investigation of which is an objective of future research.

## Data Availability

No new data were generated or analysed in support of this research.
